# Impact of periodontal mouthwashes on microbial load and periodontal indices during removable orthodontic treatment

**DOI:** 10.6026/973206300223319

**Published:** 2026-06-30

**Authors:** Shraddha S Gavade, Kiran Suradkar, Amrita Pritam, Sangita Suradkar, Naman Mittal, Sayali Mohile

**Affiliations:** 1 Department of Pediatric and Preventive Dentistry, Bharati Vidyapeeth Dental College and Hospital, Sangli, Maharashtra, India; 2 Department of Dentistry, Bharati Vidyapeeth Dental College and Hospital, Navi Mumbai, Maharashtra, India; 3 Department of Orthodontics and Dentofacial Orthopaedics, Oro Care and Orthodontic Centre, Patna, Bihar, India; 4 Department of Orthodontics, MM College of Dental Sciences and Research, Mullana, Haryana, India; 5 5Department of Pediatric and Preventive Dentistry, Swargiya Dadasaheb Kalmegh Smruti Dental College and Hospital, Nagpur, Maharashtra, India

**Keywords:** Mouthwash, orthodontic appliances, microbial load, gingival index (GI), plaque index (PI), chlorhexidine (CHX), periodontal health

## Abstract

Removable orthodontic appliances may increase plaque accumulation and microbial load, yet the effectiveness of periodontal mouthwashes remains unclear. Therefore, it is of interest to evaluate efficacy of periodontal mouthwashes on microbial load and periodontal indices in patients undergoing removable orthodontic therapy. Hence, eighty subjects were divided into groups receiving different mouthwashes, with assessment of plaque index (PI), gingival index (GI) and bleeding index (BI) at baseline and follow-up. Use of antimicrobial mouthwashes resulted in reduced microbial load and improvement in periodontal indices compared to baseline values. Thus, we support periodontal mouthwashes as effective adjuncts for maintaining oral hygiene during removable orthodontic treatment.

## Background:

The goal of orthodontic treatment is to enhance the functional occlusion and aesthetics; but in most cases, it can be difficult to maintain optimum oral hygiene, particularly in patients who are wearing removable appliances. These devices cause more retentive spaces where plaque can be deposited, which contribute to a greater number of microbial colonies and the development of periodontal inflammation is more likely [1]. The orthodontic appliances change the oral microenvironment that favors the development of the pathogenic microorganisms like *Streptococcus mutans, Porphyromonas gingivalis* and *Fusobacterium nucleatum *that cause gingivitis and periodontitis [2, 3]. Tooth brushing and flossing are still the cornerstones of the oral hygiene, yet compliance with these practices is suboptimal among the orthodontic patients, especially in teens [4]. As a result, the use of chemical plaque control agents like mouthwashes has become relevant as an adjunctive in the upkeep of periodontal health when undergoing orthodontic treatment [5]. Chlorhexidine (CHX) is the most common of these and is regarded as the gold standard because of its wide-range antimicrobial coverage as well as substantivity, which enables it to act long in the oral cavity [6]. Research has shown that chlorhexidine can be successfully used to decrease the number of bacteria and enhance health of the gingiva through inhibition of the formation of plaque and adhesion of bacteria [1]. Besides chlorhexidine, mouthwashes based on herbs and fluorides have also been tested to determine their efficiency in decreasing the amount of microbes and periodontal inflammation. The current systematic reviews indicate that chlorhexidine and herbal mouthwashes may be used effectively to reduce the gingival inflammation and plaque indices in orthodontic patients and there is no conclusive evidence of which one is better than the other in the long-term [8, 9]. Moreover, chlorhexidine usage has been found to reduce colonies of microbes and periodontal indices significantly in the initial few weeks of application [10]. The predisposition of orthodontics patients to gingival inflammation is especially associated with the amplification of plaque retention in the areas of the appliance components. Research has indicated that the number of microbes may go up tremendously during orthodontic care unless good hygiene practices are observed [11]. This highlights the importance of adjunctive chemical substances that would keep a check on microbial growth. Mouthwashes provide an effective non-invasive way of promoting oral health and decreasing chances of periodontal complications in orthodontic treatment [12]. Although there is a lot of research work on the fixed orthodontic appliances, a little research has particularly examined the effects of the mouthwashes in patients who have already undergone removable orthodontic appliances. The case of removable appliances is different since they can be removed during cleaning, but there are still compliance problems and inappropriate hygiene habits that can result in the buildup of plaque and the growth of microorganisms [13]. Thus, it is crucial to determine the efficacy of periodontal mouthwashes in this group of patients. Periodontal health is usually assessed with the help of indices like plaque index, gingival index and bleeding index that offer consistent clinical insights into inflammation and health of the oral cavity [14]. Moreover, microbiological analysis provides objective data on the change of bacteria loads, which will make it possible to conduct a complete evaluation of the treatment outcomes [15]. Therefore, it is of interest to determine the effect of periodontal mouthwashes on microbial load and periodontal indices of patients under removable orthodontic treatment.

## Materials and Methodology:

## Study design:

This study was designed as a prospective, randomized, controlled clinical study to evaluate the effect of periodontal mouthwashes on microbial load and periodontal indices during removable orthodontic treatment.

## Study setting:

The study was conducted in the Department of Orthodontics and Dentofacial Orthopedics at a tertiary dental care institution over a period of 12 months.

## Sample size:

A total of 80 subjects undergoing removable orthodontic treatment were included in the study. The sample size was determined based on previous clinical studies evaluating the effect of mouthwashes on periodontal parameters, ensuring adequate statistical power (80%) and significance level (α = 0.05).

## Study population:

Patients reporting for removable orthodontic treatment and fulfilling the inclusion criteria were recruited.

## Inclusion criteria:

[1] Patients aged 12-30 years

[2] Patients undergoing removable orthodontic appliance therapy

[3] Good general health with no systemic disease

[4] Patients with mild to moderate gingival inflammation

[5] Willingness to participate and provide informed consent

## Exclusion criteria:

[1] Patients with systemic diseases affecting periodontal health

[2] Use of antibiotics or antimicrobial mouthwashes within the last 3 months

[3] Presence of advanced periodontal disease

[4] Tobacco users

[5] Patients with fixed orthodontic appliances

## Grouping of subjects:

Participants were randomly divided into two groups (n = 40 each):

[1] Group A (Control Group): Mechanical plaque control only (tooth brushing)

[2] Group B (Test Group): Mechanical plaque control + periodontal mouthwash (e.g., 0.12% chlorhexidine / herbal mouthwash)

## Clinical parameters assessed:

The following periodontal indices were recorded at baseline, 2 weeks and 4 weeks:

[1] Plaque Index (Silness and Löe)

[2] Gingival Index (Löe and Silness)

[3] Bleeding on Probing (Sulcus Bleeding Index)

## Microbiological assessment:

[1] Plaque samples were collected from the gingival margin using sterile curettes

[2] Samples were transferred to transport media and cultured

[3] Colony-forming units (CFU) of specific bacteria such as Streptococcus mutans were assessed

[4] Quantitative microbial analysis was performed using standard microbiological techniques

## Intervention protocol:

[1] Patients in the test group were instructed to use 10-15 ml of mouthwash twice daily for 30 seconds after brushing

[2] Duration of intervention: 4 weeks

[3] Compliance was monitored through patient diaries and follow-up visits

## Statistical analysis:

Data were analyzed using SPSS software 26.0. Intragroup comparisons: Paired t-test. Intergroup comparisons: Independent t-test. Significance level set at p < 0.05 

## Results:

A total of 80 subjects undergoing removable orthodontic treatment were included in the study and equally divided into two groups: Group A (control, n=40) and Group B (mouthwash group, n=40). All participants completed the study without significant dropouts. The clinical and microbiological parameters were assessed at baseline, 2 weeks and 4 weeks. At baseline, no statistically significant difference was observed between the groups (p=0.72). At 2 weeks, Group B showed a 29.8% reduction in plaque index, which was statistically significant (p<0.001). At 4 weeks, the reduction increased to 47.8%, indicating a strong effect of mouthwash use in reducing plaque accumulation. In contrast, Group A showed only minimal reduction (≈13.5%), suggesting that mechanical cleaning alone is less effective (Table 1). Both gingival index and bleeding index showed statistically significant improvement in Group B. At 4 weeks, gingival inflammation reduced by 49.7% and bleeding index reduced by 51.1%, indicating improved periodontal health. The control group showed only modest improvements (~15-18%), reinforcing the adjunctive benefit of mouthwash (Table 1). Microbial analysis revealed a significant reduction in bacterial load in the mouthwash group. At 2 weeks, a 40.2% reduction in CFU/ml was observed, which further increased to 58.6% at 4 weeks. The control group demonstrated only a mild reduction (~15%), highlighting the effectiveness of antimicrobial mouthwashes in reducing oral microbial burden (Table 3).

## Discussion:

The current investigation determined the effectiveness of periodontal mouthwashes in reducing the microbial load and periodontal indices of patients receiving removable orthodontic therapy. The results showed that there was statistically significant decrease in accumulation of plaque, gingivitis, tendency to bleed and the microbial load in the mouthwash group than in the control group. Removable orthodontic appliances, such as removable ones, have been found to form plaque-retentive niches that promote the colonization and biofilm formation of microbes. Research has indicated a rise in the number of pathogenic microorganisms like Streptococcus mutans and Porphyromonas gingivalis in the process of orthodontic treatment, which results in gingival inflammation and periodontal loss [1]. These findings are supported by the results of the current study, where the level of microbial loads at the baseline was high in both groups. The high decrease in the plaque index was comparable to the result of recent systematic reviews indicating that antimicrobial mouthwashes are effective in reducing the amount of plaque and gingival inflammation in orthodontic patients [8]. The nearly 47.8 per cent cut in plaque index at 4 weeks in the current study is identical with past clinical trials which have proven the effectiveness of chlorhexidine in preventing plaque buildup and adhesion of bacteria [3]. Gingival health was measured by the use of gingival and bleeding indices and it was observed that the test group exhibited significant improvement in their health. The effect of gingival inflammation and bleeding reduction of about 50 per cent is similar to the outcomes of the clinical trials where a chlorhexidine mouthwash showed a significant improvement in periodontal parameters in 30 days [14]. The effect could be explained by the substantivity of chlorhexidine that enables it to exert a long-term antimicrobial effect on the tissues of the mouth. The microbiological results of the current study have shown significant decrease in the number of bacteria colonies in the mouthwash group. This is in line with past studies that have shown that mouthwashes especially chlorhexidine and fluoride-based preparations can reduce the number of microbes in orthodontics by a great margin [11]. The 58.6% reduction observed after 4 weeks shows the effectiveness of chemical plaque control measures in breaking bacterial biofilms. Interestingly, the control group recorded very little improvements with mechanical cleaning. This finding emphasizes the limitations of mechanical plaque control alone, especially in orthodontic patients where compliance is often suboptimal. Past research has also indicated that brushing in isolation might not be effective in the removal of plaque in retentional spaces formed by orthodontic appliances [4]. The other noteworthy observation is the improvement that is gradually gaining over time with maximum improvement at 4 weeks. This is a time-based tendency in line with the research that the antimicrobial activity of mouthwashes increases with increasing usage [5]. It has been demonstrated to have significant microbial load reduction especially in the short term, but long-term outcomes are variable depending on the formulation and adherence to therapy [13]. The results also lend credence to the idea that a decrease in microbial load is directly proportional to a decrease in periodontal indices. Since the bacterial biofilm is the major etiological agent in gingival inflammation, a reduction in its level results in a reduction in inflammatory response and better clinical outcomes [6]. Although the results were positive, there are some limitations to be taken into account. The time frame of the study was quite limited (4 weeks) and the long-term effects of using mouthwash were not observed. Also, the difference in patient compliance and personal oral hygiene procedures could have impacted the findings. The issue of the emergence of antimicrobial resistance when using the mouthwash over a period has also been noted in the recent literature and therefore should be used cautiously [12]. Moreover, chlorhexidine is the gold standard, but its adverse effects, including altered taste sensation and tooth stains, can reduce adherence in the long-term. Alternatives have been suggested in the form of herbal mouthwashes but they are yet to be proved in relation to their comparative effectiveness. Altogether, the current research shows that periodontal mouthwashes are useful supplements in keeping the oral cavity clean when using removable orthodontics. Their clinical relevance is demonstrated by the considerable decrease in the microbial load and the increase in the periodontal indices.

## Conclusion:

We show that periodontal mouthwashes result in significant reduction of the microbial load and increase of periodontal indices among patients subjected to removable orthodontic treatment. Adjunctive mouthwash use in conjunction with mechanical plaque control is more efficient than mechanical methods. Antimicrobial mouthwashes may be important in preventing periodontal complications in orthodontic therapy when used regularly.

## Figures and Tables

**Table 1:** Comparison of Plaque Index (PI) Between Groups

**Time Interval**	**Group A (Control) Mean ± SD**	**Group B (Mouthwash) Mean ± SD**	**% Reduction (Group B)**	**p-value**
Baseline	1.85 ± 0.32	1.88 ± 0.30	-	0.72
2 weeks	1.72 ± 0.28	1.32 ± 0.25	29.80%	<0.001*
4 weeks	1.60 ± 0.26	0.98 ± 0.22	47.80%	<0.001*

**Table 2:** Comparison of Gingival Index (GI) and Bleeding Index (BI)

**Parameter**	**Time**	**Group A Mean ± SD**	**Group B Mean ± SD**	**% Reduction (Group B) **	**p-value**
GI	Baseline	1.76 ± 0.34	1.79 ± 0.31	-	0.81
	2 weeks	1.60 ± 0.30	1.20 ± 0.27	32.90%	<0.001*
	4 weeks	1.48 ± 0.28	0.90 ± 0.24	49.70%	<0.001*
BI	Baseline	1.65 ± 0.29	1.68 ± 0.27	-	0.76
	2 weeks	1.50 ± 0.26	1.10 ± 0.25	34.50%	<0.001*
	4 weeks	1.38 ± 0.24	0.82 ± 0.21	51.10%	<0.001*

**Table 3:** Microbial Load (CFU/ml) comparison

**Time Interval**	**Group A (x10^5^CFU/ml)**	**Group B (x10^5^ CFU/ml)**	**% Reduction (Group B)**	**p-value**
Baseline	8.5 ±1.2	8.7 ±1.1	-	0.68
2 weeks	7.9 ±1.0	5.2 ±0.9	40.20%	<0.001*
